# Exploring causal correlations between inflammatory cytokines and Ménière’s disease: a Mendelian randomization

**DOI:** 10.3389/fimmu.2024.1373723

**Published:** 2024-04-29

**Authors:** SongTao Xie, RuoFeng Zhang, YuRou Tang, QingQing Dai

**Affiliations:** ^1^ Hearing Center/Hearing and Speech Laboratory, Department of Otorhinolaryngology Head and Neck Surgery, West China Hospital of Sichuan University, Chengdu, China; ^2^ Department of Hearing and Speech Rehabilitation, West China School of Clinical Medicine, Sichuan University, Chengdu, China; ^3^ Department of Otorhinolaryngology Head and Neck Surgery, West China TianFu Hospital of Sichuan University, Chengdu, China; ^4^ Otolaryngology Department, Hospital of Chengdu University of Traditional Chinese Medicine, Chengdu, China

**Keywords:** Ménière’s disease, inflammatory cytokines, causal inference, MR analysis, sensitivity

## Abstract

**Objectives:**

Previous studies have highlighted associations between certain inflammatory cytokines and Ménière’s Disease (MD), such as interleukin (IL) -13 and IL-1β. This Mendelian randomization aims to comprehensively evaluate the causal relationships between 91 inflammatory cytokines and MD.

**Methods:**

A comprehensive two-sample Mendelian randomization (MR) analysis was conducted to determine the causal association between inflammatory cytokines and MD. Utilizing publicly accessible genetic datasets, we explored causal links between 91 inflammatory cytokines and MD risk. Comprehensive sensitivity analyses were employed to assess the robustness, heterogeneity, and presence of horizontal pleiotropy in our findings.

**Results:**

Our findings indicate that MD causally influences the levels of two cytokine types: IL-10 (P=0.048, OR=0.945, 95%CI =0.894~1.000) and Neurotrophin-3 (P=0.045, OR=0954, 95%CI =0.910~0.999). Furthermore, three cytokines exhibited significant causal effects on MD: CD40L receptor (P=0.008, OR=0.865, 95%CI =0.777-0.963), Delta and Notch-like epidermal growth factor-related receptor (DNER) (P=0.010, OR=1.216, 95%CI =1.048-1.412), and STAM binding protein (P=0.044, OR=0.776, 95%CI =0.606-0.993).

**Conclusion:**

This study suggests that the CD40L receptor, DNER, and STAM binding protein could potentially serve as upstream determinants of MD. Furthermore, our results imply that when MD is regarded as the exposure variable in MR analysis, it may causally correlate with elevated levels of IL-10 and Neurotrophin-3. Using these cytokines for MD diagnosis or as potential therapeutic targets holds great clinical significance.

## Introduction

Ménière’s disease (MD) presents as a persistent inner ear condition, influenced by multiple factors, and marked by symptoms such as vertigo, sensorineural hearing loss (SNHL) predominantly affecting low-to-medium frequencies, tinnitus (ringing sensation in the ear), and aural fullness (sensation of pressure in the ear) (1, 2). Vertigo, a hallmark symptom of MD, is characterized by typical vertigo attacks lasting from 20 minutes to one day (2). As outlined in clinical guidelines (1), MD diagnosis is primarily clinical, where unilateral ear symptoms persist over several decades. MD attacks occur randomly and episodically, averaging approximately 6-11 per year, interspersed with remission periods lasting months to years. Consequently, the diagnosis of MD is often a prolonged process, evolving over months or even years due to the complexities of its clinical presentation. Precise cause of MD (3, 4) remains uncertain, although it has been associated with changes in the fluid volumes within the inner ear, known as endolymphatic hydrops (EH), a characteristic feature of the condition and can be confirmed through postmortem examination. Though the exact origins and mechanisms of MD remain unclear, there is substantial evidence from various studies indicating the significant involvement of inflammation in the onset and recurrence of the disease (5).

The inflammatory state in MD patients is summarized as follows: MD as an endophenotype in bilateral MD associated with the allelic variant rs4947296 and nuclear factor-kappa B (NF-κB)-mediated inflammation; The role of cytokines, especially interleukin (IL) -1βand tumor necrosis factor-α (TNF-α), in identifying a subset of patients with autoinflammation; and the potential of cytokines as biomarkers for distinguishing MD from vestibular migraine (5, 6). Previous research has also closely linked cytokines such as IL-13 and IL-1β with MD (7, 8). However, question of whether systemic inflammation is a cause or a consequence of MD remains subject to debate. This uncertainty arises from factors as disease progression, subsequent infection, medication usage after MD onset, and potential biases in observational studies due to unanticipated confounding variables or reverse causation. These complexities make it challenging to establish definitive causal relationships (9).

Mendelian randomization (MR) serves as an analytical approach used to infer the causal effect of an exposure on an outcome, leveraging genetic variations in non-experimental data (10). Due to the random assignment of alleles during meiosis, MR can effectively mitigate the influence of both conventional confounding variables and reverse causation, thereby offering robust evidence for causal inference (11). Through two-sample MR analysis, researchers can evaluate instrument–exposure and instrument-outcome relations in two separate population samples, enhancing the applicability and effectiveness of the test (12). In this study, we initially identified valid genetic variants from published genome-wide association study (GWAS) summary data of 91 inflammatory cytokines to investigate their correlations with MD. Subsequently, we examined the direction of causation by reversing the exposure and outcome.

## Methods

This MR analysis utilized previously published GWAS summary statistics. Ethical approval from the institutional review boards and written informed consent from participants were obtained for the separate studies contributing to the GWAS data. Additional ethical approval or informed consent was deemed unnecessary. The STROBE-MR checklist has been thoroughly reviewed and is provided as **Supplementary Data** (13).

### MR assumptions

**Figure 1:**
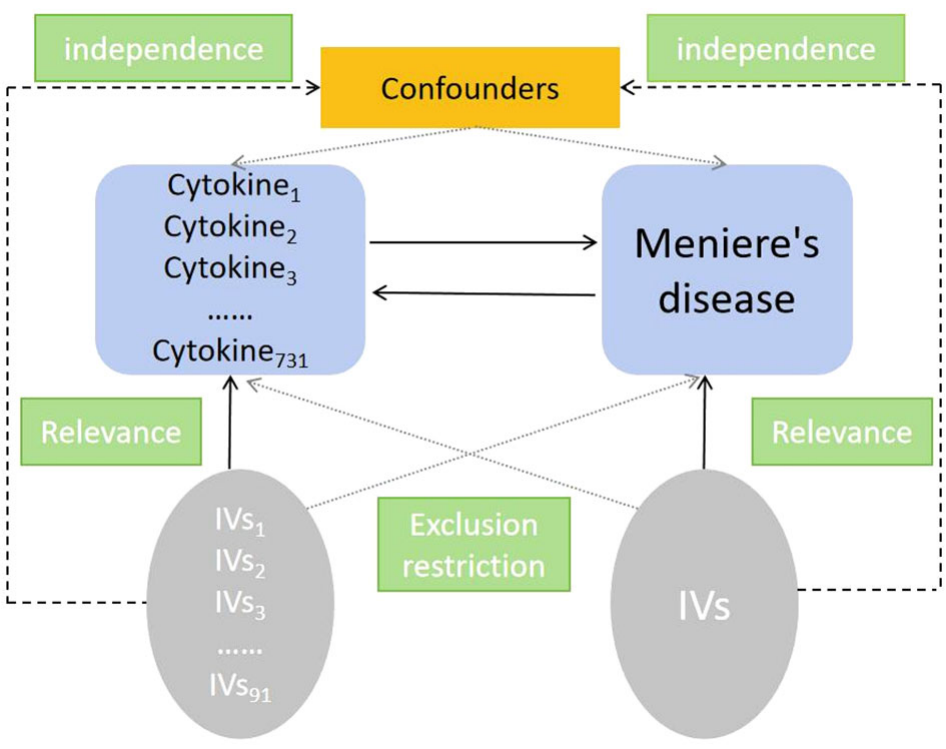
Study Design Schematic for Bidirectional MR Analysis. In this study, significant instrumental variables were identified for 91 inflammatory cytokines and MD, enabling exploration of bidirectional causal relationships. The schematic illustrates the three core assumptions of MR analysis, exclusion restriction, independence, relevance, depicted in a causal directed acyclic graph.

MR analysis relies on three fundamental assumptions: exclusion restriction, independence, relevance (14). It is hypothesized that the identified genetic variants are linked to the risk factor (relevance) but not influenced by any confounding factors in the association between the risk factor and the outcome (independence). Furthermore, it is suggested that these variants solely affect the outcome through the specific risk factor of interest (exclusion restriction). In this two-way study, two GWASs were utilized to identify significant single nucleotide polymorphisms (SNPs) associated with 91 inflammatory cytokines and Ménière’s disease (**Figure 1**).

### GWAS data sources for MD

Data utilized for this MD analysis was obtained from FinnGen, an extensive public-private research initiative. FinnGen integrates imputed genotype data from both newly collected and legacy samples from the Finnish biobank with digital health record data from the Finnish Health Registry, aiming to offer novel insights into disease genetics (15). MD Dataset comprised 2746 cases and 359,094 controls of European ancestry(https://r9.risteys.finngen.fi/endpoints/H8_MENIERE). The diagnostic criteria was ICD-10/H81.0.

### Immunity-wide GWAS data sources

For inflammatory cytokines, we used data from a study identifying genome variant associations with 91 plasma proteins, being measured by Olink Target Inflammation panel across 11 cohorts, encompassing a total of 14,824 participants of European-ancestry. GWAS summary statistics from this study were previously published in Journal of Nat Immunol (16).

### Selection of instrumental variables

**Figure 2:**
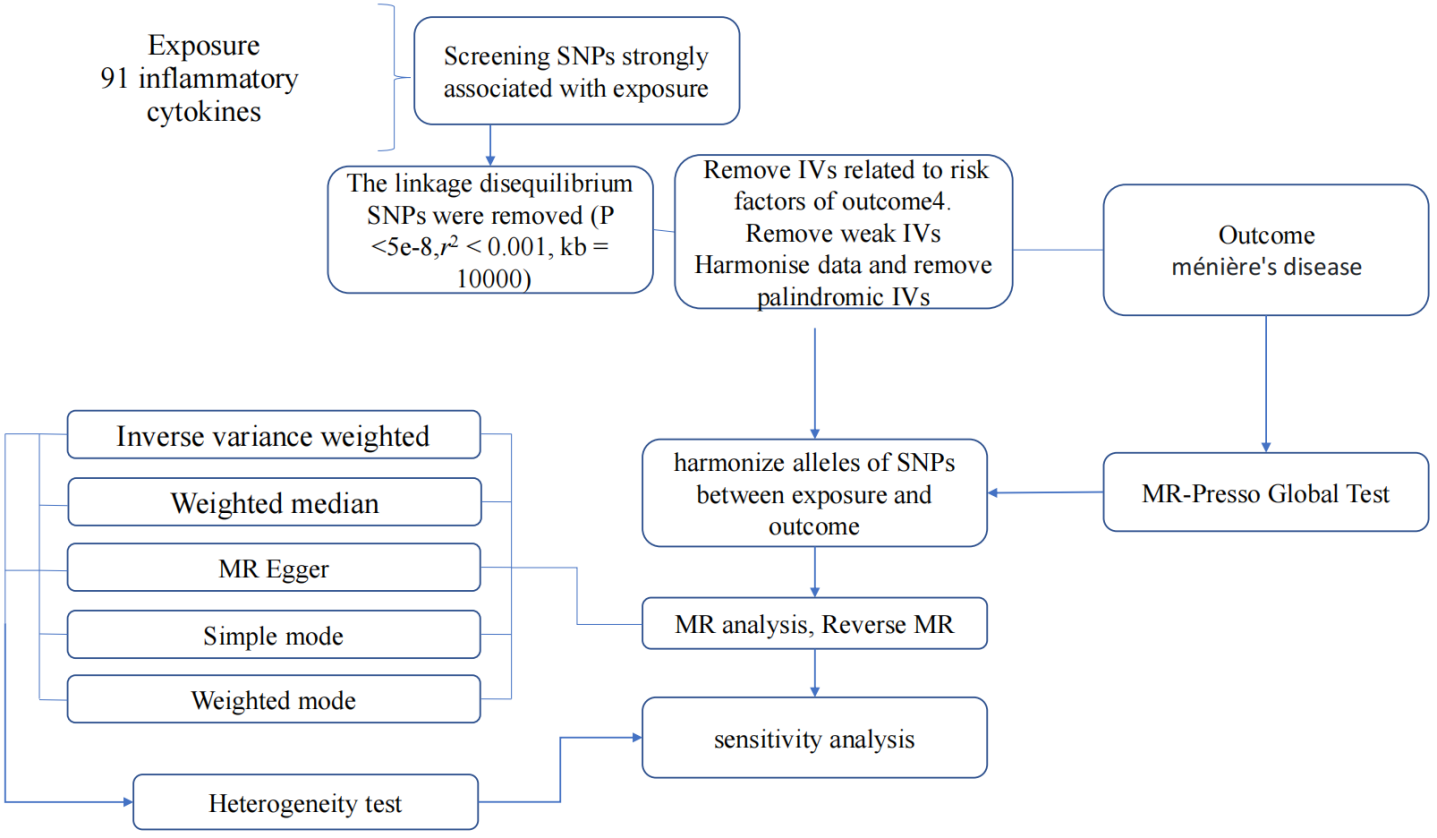
Flow diagram depicting the quality control process for IVs and the overall MR analysis process. SNPs, single-nucleotide polymorphisms; IVW, inverse variance weighted; MR, Mendelian Randomization; MR Presso, Mendelian Randomization Pleiotropy RESidual Sum and Outlier.

In summary, our study employed a MR design with 91 inflammatory cytokines as the exposure and MD as the outcome. To ensure the credibility and accuracy regarding the causal relationship conclusions between inflammatory cytokines and MD risk, several quality control steps were implemented to select optimal IVs. The entire process is illustrated in **Figure 2**.

### Statistical analysis

Initially, we established a genome-wide significant threshold (p <5 × 10^-8^) for selecting SNPs strongly related to MD and inflammatory cytokines. To address the limited available SNPs for cytokines as exposures, a more stringent cutoff (p <5 × 10^-6^) was initially employed. If an adequate number of SNPs were still not identified, the threshold was further adjusted. Ultimately, for our MR analysis, a threshold of p <1 × 10^-5^ was established. Furthermore, to mitigate issues related to linkage disequilibrium, the identified SNPs were subjected to clumping (kb = 10,000, r^2 =^ 0.001). SNPs displaying palindromic characteristics were excluded due to uncertainty regarding their alignment in the same direction for exposure and outcome in GWASs of systemic inflammatory regulators. The strength of the instruments was evaluated using the F-statistic to address potential biases arising from weak instruments (17, 18). SNPs with an F-value below 10 were deemed weak instruments and were therefore excluded from the analysis. Finally, in instances where SNPs were unavailable in the outcome summary, they were substituted with proxy SNPs (R^2^ > 0.9) sourced from LDlink (19).

### MR analysis

We performed MR analysis to investigate the causal connections between 91 inflammatory cytokines and MD using various established MR methodologies. Recognizing that not all genetic variants may serve as valid instrumental variables, we employed several robust methods. These methods, including MR-Egger regression, MR-Pleiotropy RESidual Sum and Outlier (MR-PRESSO) method, weighted median (WM), weighted mode, and inverse variance weighting (IVW)., are grounded on different assumptions.

IVW method adopts a meta-analysis strategy to aggregate Wald estimates for each SNP, generating overall estimates of the effect of MD on cytokines (20). In instances where the IV2 assumption (absence of horizontal pleiotropy) remains unviolated or where horizontal pleiotropy is balanced, IVW linear regression yields an unbiased causal estimate (21). Fixed/random effects IVW approaches are available. In the presence of notable heterogeneity (P < 0.05), a random-effects IVW model is utilized. MR-PRESSO serves as a tool for identifying and rectifying outliers within IVW linear regression. Comprising three main components, MR-PRESSO includes: (a) identification of horizontal pleiotropy through the MR-PRESSO global test, (b) adjustment for horizontal pleiotropy by removing outliers via the MR-PRESSO outlier test, and (c) evaluation of significant variations in causal estimates before and after outlier correction through the MR-PRESSO distortion test. For the MR-PRESSO outlier test to be applicable, it necessitates a minimum of 50% of the variants being deemed valid instruments, exhibiting balanced pleiotropy. Moreover, it relies on the condition of Instrument Strength Independent of Direct Effect (InSIDE), stipulating that the effects of instruments on exposure and pleiotropy remain uncorrelated (22). The mode-based method groups SNPs based on their similarity in causal effects and provides the causal effect estimate based on the cluster containing the highest number of SNPs. Even if the majority of instruments are deemed invalid, the causal estimate from the mode-based estimator remains unbiased as long as the SNPs contributing to the largest cluster are considered valid instruments (23). Even when as much as 50% of SNPs are considered invalid IVs, potentially due to pleiotropy, the median-based approach ensures an unbiased estimate of the causal effect, even in the presence of unbalanced horizontal pleiotropy (24). When a specific direction of horizontal pleiotropic effect is present, imposing a constraint for the slope to pass through zero can lead to bias. Egger regression, which permits the intercept to pass through a value other than zero, helps alleviate this constraint. MR-Egger regression, under the assumption of InSIDE, conducts a weighted linear regression of the outcome coefficients on the exposure coefficients (25). Under the assumption of InSIDE, it provides a reliable test for the null causal hypothesis and a consistent estimate of the causal effect, even in scenarios where all genetic variants are deemed invalid IVs (26). Nevertheless, MR-Egger estimates can be imprecise and susceptible to significant influence from outlier genetic variants. In contrast, WM estimate, which doesn’t necessitate the InSIDE assumption, has been demonstrated to offer notable advantages over MR-Egger. Specifically, it exhibits enhanced power in detecting causal effects and demonstrates lower type I error rates (24). In scenarios where the InSIDE assumption holds true and the proportion of horizontal pleiotropic variants is minimal (≤10%), the MR-PRESSO outlier adjustment yields a less biased causal estimate with improved precision (lower standard deviation) compared to MR-Egger. Conversely, when the percentage of horizontal pleiotropic variants is substantial (≥ 50%), the reverse trend is observed (22). When assessing causal estimates, the weighted median method displays reduced bias compared to the MR-PRESSO outlier test. However, its precision is also diminished, especially evident when the proportion of horizontal pleiotropic variants is less than 50% (22). In instances where estimates from various methods yield inconclusive results, significance is attributed to the association between exposure and outcome phenotype if the adjusted P value is less than 0.01 (Bonferroni correction for multiple testing), denoting P value <0.05/5 = 0.01. Statistical analyses were performed using specific R packages: TwoSampleMR (17) and MR-PRESSO.

### Pleiotropy and sensitivity analysis

We employed MR-Egger regression to evaluate potential pleiotropic effects of the SNPs utilized as IVs. The intercept term in MR-Egger regression serves as a valuable indicator to assess whether directional horizontal pleiotropy influences the outcomes of the MR analysis (26). In MR-PRESSO analysis, efforts are made to mitigate heterogeneity in estimating the causal effect by eliminating SNPs that contribute disproportionately to the observed heterogeneity. The MR-PRESSO analysis utilized 1000 distributions. Both the IVW method and MR-Egger regression were employed to detect heterogeneity, quantified using the Cochran Q statistic, with significance defined as a P value <0.05. “Leave-one-out” sensitivity analysis was conducted to identify potentially influential SNPs, wherein MR was performed iteratively with each SNP omitted in turn. If significant horizontal pleiotropy was detected by MR-PRESSO analysis, outlier variants (with P values below the threshold in the MR-PRESSO outlier test) were removed, followed by repeating the MR analysis. If heterogeneity persisted after the MR-PRESSO outlier removal step, further analysis was conducted by excluding SNPs with P values less than 1 in the MR-PRESSO outlier test. If potentially influential SNPs were identified in the “leave-one-out” sensitivity analysis, caution was exercised in drawing conclusions.

### Reverse MR analysis

To investigate if MD causally impacts specific inflammatory cytokines, we also conducted a reverse MR analysis, with MD as exposure and 91 inflammatory cytokines as outcomes.

## Results

### Exploring causal effect of inflammatory cytokines on MD

**Figure 3:**
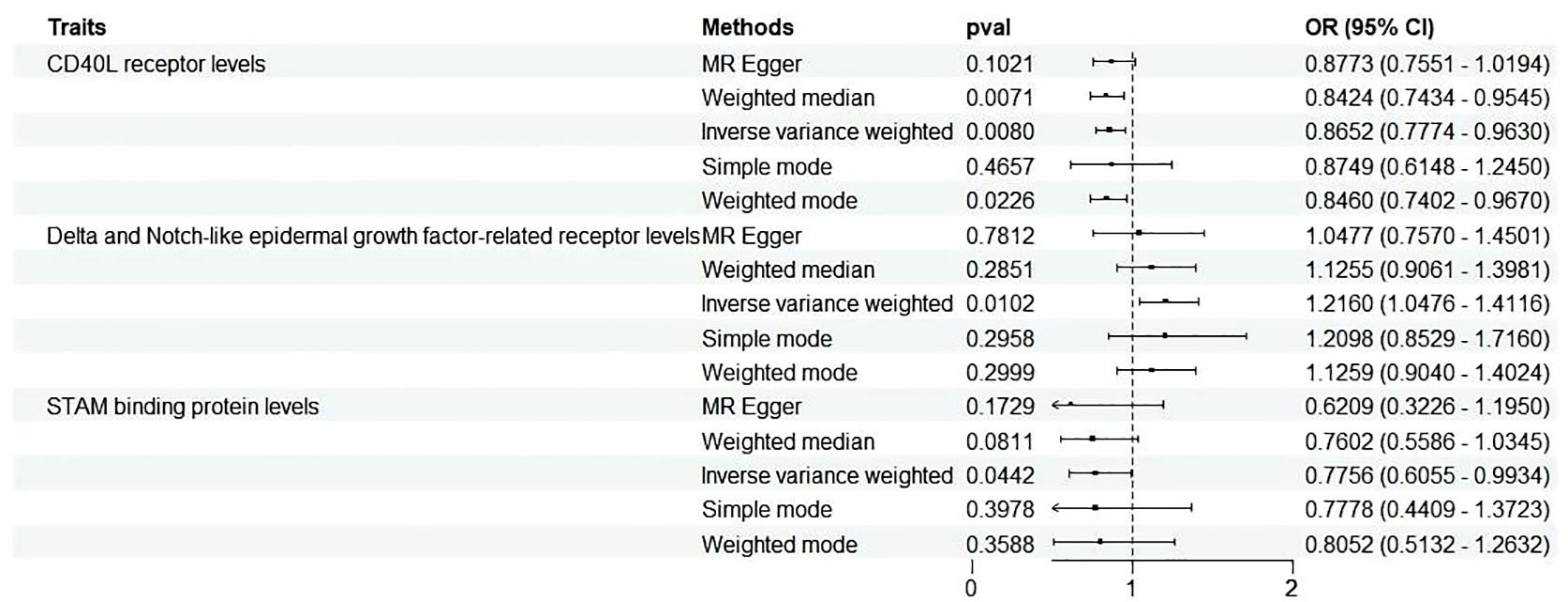
Forest plots illustrated the causal associations between inflammatory cytokines traits and MD using various methods. IVW, inverse variance weighting; CI, confidence interval. Q1 p-value, p-value of Q test from MR-Egger method; Q2 p-value, p-value of Q test from IVW method; Q, Cochran Q statistics.

**Figure 4:**
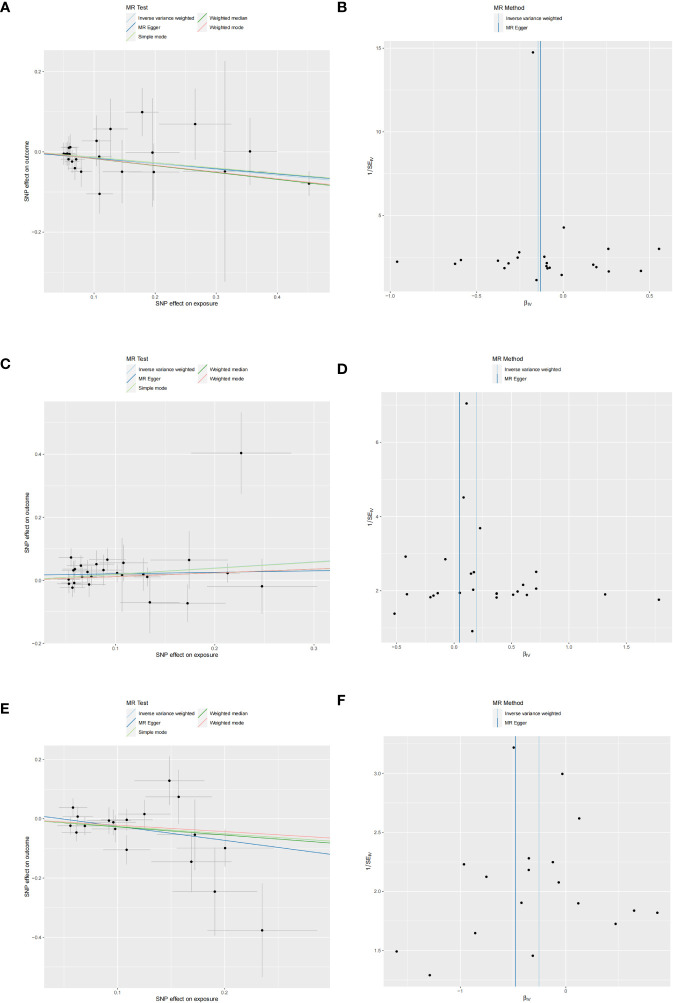
Scatter and funnel plots of MR analyses for CD40L receptor levels, Notch-like epidermal growth factor-related receptor levels, and STAM binding protein levels in MD. **(A, C, E)** Individual IV associations with cytokine risk are displayed versus individual IV associations with MD in black dots. The estimated causal effect of MR methods is depicted by the slope of lines in funnel plots, where 95% CI of the odds ratio for each IV is indicated by vertical and horizontal lines. **(B, D, F)** The funnel plots illustrate IVW MR estimate of each cytokine SNP with MD versus 1/standard error (1/SEIV).

With the threshold set at P<1×10^-5^, three cytokines out of 91 demonstrated a positive causal relationship with MD (**Figures 3**, **4**). F-statistics for each exceeded 10, indicating a minimal likelihood of weak instrument bias (**Supplementary Table S1**).

Three cytokines identified are as follows: CD40L receptor (P=0.008, OR=0.865, 95%CI=0.777-0.963), DNER (P=0.010, OR=1.216, 95%CI=1.048-1.412), and STAM binding protein (P=0.044, OR=0.776, 95%CI =0.606-0.993) (**Figure 3**). Scatter plots and funnel plots depicting MR analyses for these cytokines in MD are presented in **Figure 4**.

No heterogeneity was detected in associations from three cytokine types, as evidenced by Cochrane’s Q tests (both p-value of Q test from MR-Egger method and p-value of Q test from IVW method >0.05), while MR-PRESSO method identified no outlier SNPs. Additionally, the MR-Egger intercept disclosed no evidence of directional pleiotropy (p-value >0.05) (**Table 1**). SNP details are in **Supplementary Table S1**, while forest plots and leave-one-out sensitivity analyses of MR for the three cytokines in MD are in **Supplementary Figures S1, S2**.

**Table 1:** Heterogeneity and horizontal pleiotropy tests of inflammatory cytokines traits on MD.

Exposures	Heterogeneity tests		Test for directional horizontal pleiotropy			MR-PRESSO Global Test p-value	
	Q1 p-value	Q2 p-value	egger_intercept	se	p-value	Raw	Outlier-corrected
The causal effect of inflammatory cytokines on MD							
CD40L receptor levels	0.821	0.858	-0.003	0.011	0.798	0.861	NA
Notch-like epidermal growth factor-related receptor levels	0.399	0.397	0.016	0.016	0.322	0.432	NA
STAM binding protein levels	0.172	0.190	0.022	0.031	0.481	0.226	NA
The causal effect of Ménière’s disease onset on inflammatory cytokines							
Interleukin-10 levels	0.220	0.148	0.021	0.014	0.169	0.182	NA
Neurotrophin-3 levels	0.384	0.454	-0.005	0.013	0.707	0.473	NA

### Exploration of the causal effect of MD onset on inflammatory cytokines

**Figure 5:**
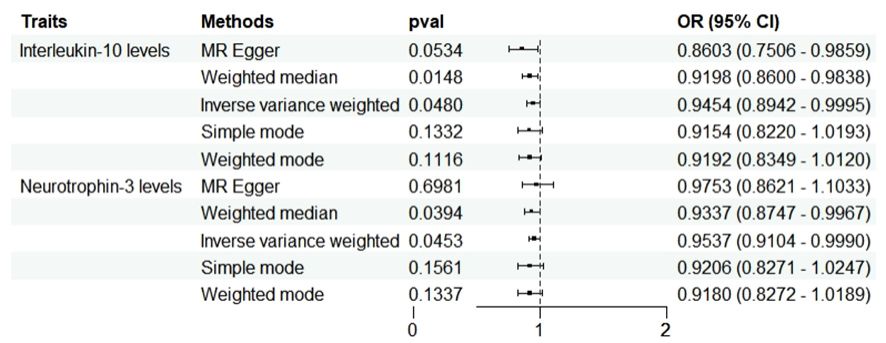
Forest plots showed the causal associations between MD and inflammatory cytokines traits by using different methods. CI, confidence interval; OR, Odds Ratio.

In the reverse analysis, with the threshold set to P <5 × 10^-6^, MD exhibited a negative causal relationship with IL-10 (P=0.048, OR=0.945, 95%CI =0.894-1.000) and Neurotrophin-3 (P=0.045, OR=0954, 95%CI =0.910-0.999) (**Figure 5**). These findings suggest that MD may induce certain protective responses, potentially involving variations in inflammation and changes in neuro-factors associated with EH pathologically.

**Figure 6:**
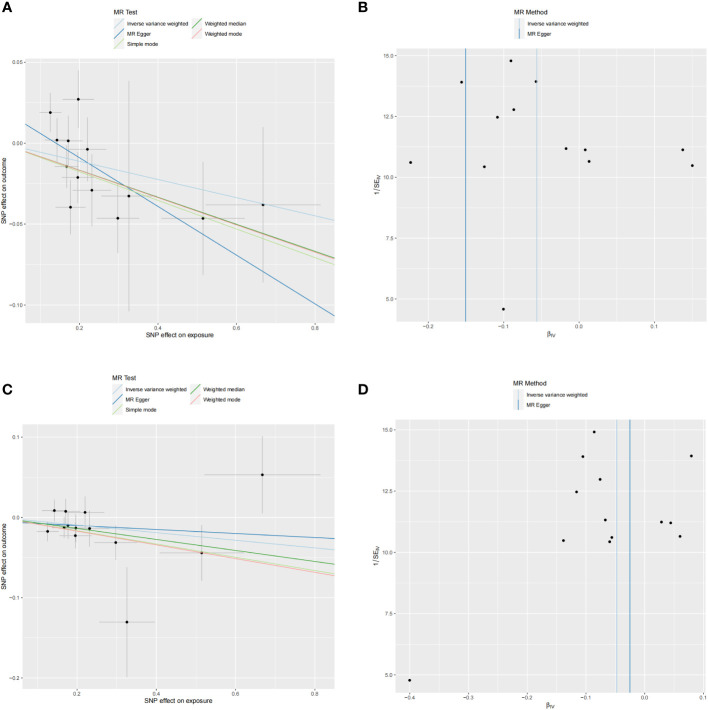
Scatter and funnel plots of MR analyses for MD on IL-10 and Neurotrophin-3 levels. **(A, C)** Individual IV associations with cytokine risk are displayed versus the ones with MD in black dots. The 95%CI of odd ratio for each IV is denoted by vertical and horizontal lines. The slope of the lines represents the estimated causal effect of corresponding MR methods. **(B, D)** The funnel plots show the IVW MR estimate of each cytokine SNP with MD versus 1/standard error (1/SEIV).

No heterogeneity was observed in the associations of IL-10 (P_MR-Egger_=0.220; P_ivw_= 0.148) and Neurotrophin-3 (P_MR-Egger_=0.384; P_ivw_=0.454), as assessed by Cochrane’,s Q test, and the MR-PRESSO method detected no outlier SNPs. Furthermore, the MR-Egger intercept showed no evidence of directional pleiotropy (p=0.169; p=0.707) (**Table 1**). Scatter and funnel plots of MR analyses for these cytokines in MD are exhibited in **Figure 6**.

SNP details are listed in **Supplementary Table S1**. Forest plots and leave-one-out sensitivity analyses of MR for IL-10 and Neurotrophin-3 levels in MD are available in **Supplementary Figures S3, S4**.

## Discussion

Utilizing large public genetic datasets, we investigated the causal links between 91 inflammatory cytokine traits and MD. This study represents the first to conduct MR analysis investigating the causal relationships of multiple cytokine types with MD. Our findings revealed that MD causally influences two inflammatory cytokines, while three cytokines significantly impact MD.

**Figure 7:**
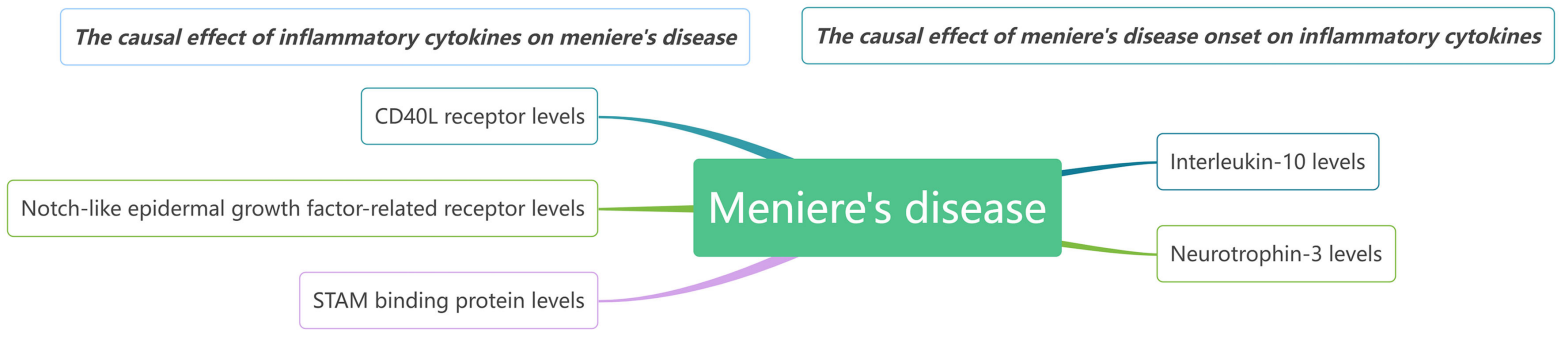
The causal relationships between inflammatory cytokines and MD by MR analysis.

Our findings suggest that CD40L receptor, DNER, and STAM binding protein may serve as upstream contributors to MD. Conversely, when MD is considered the exposure in MR, it appears that it causally increase levels of IL-10 and Neurotrophin-3. We observed no reverse causality between any single biomarker and MD. It seems that several biomarkers might trigger MD onset, while others, such as certain inflammatory regulators, are likely implicated in disease progression. A comprehensive network model illustrating these relationships is presented in **Figure 7**.

The primary histopathological alteration observed in MD is EH. This discovery dates back to 1938 when Hallpike and Cairns first reported EH in temporal bone samples from two MD patients (27). Subsequently, in 1943, Altman and Flower proposed that disorders in endolymph production and absorption contribute to the onset of MD (28). However, doubts arose regarding the association between EH and MD when Merchant et al. (29) found that only 51 out of 79 MD patients with endolymphatic hydropathy exhibited symptoms of MD. Consequently, EH was deemed to be merely a direct contributor to cochlear vestibular symptoms in MD patients, rather than a definitive pathological mechanism. Despite these challenges to the EH theory, it remains a credible and compelling potential pathogenic mechanism for MD. Various theories have emerged regarding the etiology of EH (30), including immune damage (including autoimmune disease), inner ear ischemia, and calcium ion overload. Each of these theories provides distinct explanations for the potential pathological mechanism underlying EH.

In recent years, scholars have increasingly focused on the relationship between MD and the immune system. Some studies (31) have proposed several main reasons for the association between the immune system and the incidence of MD. (1) Porous blood supply in the endolymphatic sac may introduce immune antigens into the body, leading to mast cell degranulation and inflammatory reactions; (2) Circulating immune complexes can enter the endolymphatic sac circulation and vasculature, triggering inflammation and disrupting fluid balance; (3) Viral antigen immunity. Some authors (32) studied the relationship between known immunogenic proteins and proteins in the inner ear by assessing their sequence similarity. Their findings revealed the presence of 68~72kDa inner ear immune antigens in patients with sensorineural hearing loss, which were associated with candidate immune epitopes of Hsp70 and choline transporter-like protein 2, suggesting that MD may be an immune-mediated disease. Additionally, it was noted that MD patients were three times more likely to develop allergic diseases. The screening value of allergen-specific IgE antibodies against wheat was found to be over twice that of normal individuals, while the number of immune complexes in the inner ear and the levels of anti-herpes IgG in lymphocytes and outer lymph were significantly elevated. For the above wheat allergen specific detection, a MD case was reported (33) depicting a 63-year-old female patient. She also experienced knuckle rheumatism, constipation and recurrent abdominal pain. Considering the possible correlation between gluten sensitivity and the onset of MD, a gluten-free diet was attempted during treatment, yielding noticeable therapeutic effects. Additionally, while the autoimmune background of immune responses in MD may be suspected, it’s crucial not to overlook the clinical efficacy of corticosteroids in MD, which are commonly used for autoimmune disorders in everyday clinical practice (34, 35).

IgE stands as a significant immunoglobulin in the immune system, playing a pivotal role in allergic reactions and immune-related diseases. In allergic responses, IgE binds to the allergens, triggering activation of other immune cells and consequent allergic symptoms. It mediates immune responses such as inflammation and immune regulation in non-allergic diseases. In the context of MD, IgE, as an allergy mediator, may exacerbate MD symptoms (36). Recent investigations have extensively explored the correlations between MD and a wide array of immune inflammation-related cytokines. Meta-analyses have underscored notable associations between MD and conditions like airway allergic disease and autoimmune thyroid disease (37), suggesting that allergic reactions could potentially trigger EH, consequently leading to MD (38, 39). Notably, MD patients exhibit a distinctive cytokine profile characterized by Th17/Treg imbalance, notably elevated Treg and Treg/Th17 levels in individuals with allergies compared to controls (37). Aberrant functions of key immune cells like T-lymphocytes, which produce regulatory or inflammatory cytokines, have also been elucidated in systemic lupus erythematosus (SLE) (39). In line with other autoimmune disorders, elevated NaCl concentrations have been associated with the release of pro-inflammatory cytokines such as IL-1β and IL-6 in MD (39). Additionally, certain cytokines like IgE, IL-4, IL-5, IL-10, and IL-13 are upregulated in certain MD patients (28), with IL-4 known to regulate CD23 expression in B cells and promote IgE production (40).

Despite these discoveries, establishing causal connections between MD and key cytokines remain challenging due to limitations inherent in classical epidemiology. Classical epidemiological methods often enhance statistical power in identifying correlations but contribute minimally to theoretical understanding of causation (41). Interpreting a relationship between exposure and disease outcome in observational data as causal relies on assumptions that are often unattainable, such as the absence of unexpected confounding and reverse causation (42). Therefore, the observed increase in inflammatory cytokines among MD patients could signify various factors, including disease’s cause, a treatment side effect, an underlying infection, or other concurrent pathological immune responses, distinctions not discernable in standard observational studies. Our cytokine MR study illuminates the immune system’s close ties to EH in MD.

The primary conclusion from this MR study is that the CD40L receptor, DNER, and STAM binding protein might contribute in the onset of MD. CD40, belonging to the TNF receptor superfamily, is expressed on various cell types, both immune and non-immune. Its interaction with CD40L, primarily found on activated CD4+ T cells, is essential for the formation of germinal centers and the production of class-switched antibodies. CD40 expression on non-hematopoietic cells can also engage CD40L, leading to a pro-inflammatory response (36), being extensively studied in autoimmune diseases (43, 44). DNER, a neuron-specific transmembrane protein with extracellular EGF-like repeats (44), has been demonstrated to facilitate prostate cancer advancement and enhance PC-3 cell proliferation by modulating the core genes associated with cancer stem cells (45). DNER was additionally discovered to be upregulated in gastric cancer, and its suppression led to inhibited growth and metastasis (46), also contributing to breast cancer progression by promoting stemness (47). STAM binding protein, or STAMBP, is a JAMM-family deubiquitinating enzyme with microtubule-interacting/transport and STAM-binding domains. Its role in regulating melanoma metastasis via SLUG underscores its potential as a therapeutic target (48). STAMBP’s role in promoting malignancy in pancreatic cancer cells and its involvement in the regulatory mechanism of hsa_circ_0007334 has also been reported (49). Despite their roles in autoimmune diseases and cancers, the involvement of these proteins in MD remains largely unexplored. Further research is necessary to elucidate their potential functions in MD pathogenesis, requiring more comprehensive data to clarify their relationship with the disease.

Regarding IL-10 and Neurotrophin-3, MD could potentially elevate their levels through causal pathways. IL-10, a potent anti-inflammatory and immunosuppressive cytokine, is produced by various cells of both innate and adaptive immunity, including dendritic cells, macrophages, mast cells, natural killer cells, eosinophils, neutrophils, B cells, CD8+ T cells, and TH1, TH2, and TH17, as well as regulatory T cells (50). Numerous studies have highlighted its significance in disease progression. Previous research on MD patients suggested that an imbalance of IL-17/IL-10 plays a significant role in the onset of the disease (51), with IL-10 levels being upregulated in some MD patients (7). Our findings align with these clinical studies. However, the specific role of the IL-10 cytokine in initiating immune responses and its subsequent impact in the disease context remain open questions for future research.

Neurotrophin-3 holds promise as a pharmacological target for mood disorders due to its influence on monoamine neurotransmitters, regulation of synaptic plasticity and neurogenesis, enhancement of brain-derived neurotrophic factor (BDNF) signaling, and modulation of the hypothalamic-pituitary-adrenal (HPA) axis, which is implicated in the neurobiological processes underlying mood and anxiety disorders (52). Depression is reported in nearly 50 percent of MD cases (53). Observational studies indicate that patients with MD have an increased depression risk, while individuals with depression are more prone to developing MD (54). The onset and progression of mood disorders and MD are intricately linked (55–58). While Neurotrophin-3 holds promise as a pharmacological target for mood disorders, its function in MD remains unclear. Hence, further research is warranted to explore the prognostic and diagnostic value of Neurotrophin-3 in MD, its role in pathogenesis, and the efficacy of Neurotrophin-3-targeted treatments, leveraging insights from previous studies on mood disorders and this MR study. Investigating the potential interplay between Neurotrophin-3 and MD, as well as the specific role of Neurotrophin-3 cytokines in immune initiation and disease progression, are crucial areas for future investigation. Notably, discussions in the literature regarding targeted drugs like IL-6 targeted Siltuximab for autoimmune/autoinflammatory MD suggest the feasibility of Neurotrophin-3-targeted therapies for MD (59).

If immune regulators demonstrate specific exposure-outcome linkages with disease onset, their therapeutic potential is promising. It is the first MR study to evaluate the causal relationship between MD and 91 inflammatory cytokines. However, several **limitations** should be acknowledged. Firstly, due to constraints in MR analysis, thorough examination of the second and third assumptions was not possible, potentially introducing biases. Secondly, the absence of individual data precluded further stratified population analyses. Thirdly, as the study relied on a European database, the findings may not be generalizable to other ethnic groups, thus limiting the applicability of our results. Finally, a looser threshold was used to evaluate results, which might have increased false positives but also allowed for a more comprehensive assessment of the strong associations between cytokines profiles and MD. Further research is warranted to validate our findings and to consider their application in clinical diagnostics and therapeutic strategies.

## Conclusion

In summary, our comprehensive bidirectional MR analysis has elucidated causal associations between several inflammatory cytokines and MD, highlighting the intricate interplay between the immune system and MD. Our research opens new avenues for exploring MD biological mechanisms, potentially leading to earlier diagnosis and the development of intervention and treatment strategies. Our findings enhance the understanding of inflammatory cytokines in MD, offering valuable insights for its diagnosis, prevention and therapy.

## Data availability statement

The raw data supporting the conclusions of this article will be made available by the authors, without undue reservation.

## Author contributions

SX: Data curation, Investigation, Project administration, Writing – original draft. RZ: Data curation, Methodology, Resources, Validation, Writing – review & editing. YT: Investigation, Project administration, Resources, Software, Writing – review & editing. QD: Conceptualization, Funding acquisition, Investigation, Methodology, Supervision, Writing – original draft, Writing – review & editing.
