# Supplementary material for: Exploring causal correlations between inflammatory cytokines and Ménière’s disease: a Mendelian randomization
Source: Front Immunol. 2024 Apr 29;15:1373723. doi: 10.3389/fimmu.2024.1373723 (PMC11089180; doi:10.3389/fimmu.2024.1373723)

***Supplementary Materials***

***Supplementary material-table***

Table S1. SNP information on MR results of inflammatory cytokines on Meniere's disease.

| **The causal effect of inflammatory cytokines on meniere's disease** | | | | | | | | | | |
| --- | --- | --- | --- | --- | --- | --- | --- | --- | --- | --- |
| **exposures** | **nSNP** | **SNP** | **effect_allele.exposure** | **other_allele.exposure** | **beta.exposure** | **eaf.exposure** | **se.exposure** | **pval.exposure** | **R2** | **F** |
| CD40L receptor levels | 23 | rs117191103 | T | C | 0.3553 | 0.0208 | 0.0437 | 4.28E-16 | 0.00446585 | 66.09499799 |
|  |  | rs11755766 | T | C | -0.0504 | 0.5049 | 0.0112 | 6.80E-06 | 0.001372393 | 20.24725144 |
|  |  | rs12152184 | A | G | 0.1085 | 0.8849 | 0.0205 | 1.21E-07 | 0.002253617 | 28.00797515 |
|  |  | rs12500563 | T | G | 0.3143 | 0.9875 | 0.0687 | 4.76E-06 | 0.001935136 | 20.92641426 |
|  |  | rs138897138 | A | G | 0.109 | 0.914 | 0.0216 | 4.50E-07 | 0.001779101 | 25.46154177 |
|  |  | rs144949926 | A | G | -0.2655 | 0.9784 | 0.0591 | 7.04E-06 | 0.001410489 | 20.1787051 |
|  |  | rs149900704 | A | G | -0.1956 | 0.0203 | 0.0442 | 9.63E-06 | 0.001327561 | 19.58096986 |
|  |  | rs17372980 | T | C | -0.0611 | 0.7421 | 0.0134 | 5.12E-06 | 0.001453014 | 20.78796744 |
|  |  | rs187640246 | T | C | -0.2717 | 0.0134 | 0.0604 | 6.85E-06 | 0.001579358 | 20.23193837 |
|  |  | rs1883832 | T | C | -0.4519 | 0.2575 | 0.0126 | 1.00E-200 | 0.080282082 | 1286.129338 |
|  |  | rs34755276 | A | G | 0.0587 | 0.3475 | 0.0131 | 7.43E-06 | 0.001616235 | 20.07537077 |
|  |  | rs35864161 | T | G | -0.1269 | 0.9543 | 0.0284 | 7.88E-06 | 0.001353158 | 19.96308302 |
|  |  | rs3967170 | A | G | 0.0789 | 0.1353 | 0.017 | 3.46E-06 | 0.001461113 | 21.53759253 |
|  |  | rs404842 | A | G | -0.0599 | 0.6433 | 0.0123 | 1.12E-06 | 0.001657227 | 23.71278818 |
|  |  | rs4278412 | C | G | 0.1978 | 0.0203 | 0.0413 | 1.67E-06 | 0.001555325 | 22.93472198 |
|  |  | rs4664334 | T | C | 0.0541 | 0.5003 | 0.0118 | 4.55E-06 | 0.001468996 | 21.01695139 |
|  |  | rs7250371 | T | C | 0.0686 | 0.3648 | 0.0116 | 3.34E-09 | 0.002369927 | 34.96819776 |
|  |  | rs73128569 | A | T | -0.1788 | 0.9406 | 0.0268 | 2.53E-11 | 0.003105583 | 44.50457367 |
|  |  | rs74794857 | T | C | 0.0707 | 0.4128 | 0.0138 | 3.00E-07 | 0.002226923 | 26.24259566 |
|  |  | rs75626299 | C | G | 0.1458 | 0.9638 | 0.0316 | 3.95E-06 | 0.001443344 | 21.28540428 |
|  |  | rs7798473 | A | C | -0.1041 | 0.0609 | 0.0235 | 9.43E-06 | 0.001330047 | 19.6203513 |
|  |  | rs8024256 | A | G | -0.0562 | 0.3092 | 0.0121 | 3.41E-06 | 0.001461797 | 21.56964231 |
|  |  | rs9526031 | A | C | 0.064 | 0.4882 | 0.012 | 9.64E-08 | 0.001986837 | 28.44046286 |
|  |  | rs9993874 | A | G | -0.0582 | 0.4883 | 0.0125 | 3.22E-06 | 0.00174478 | 21.67484034 |
| Notch-like epidermal growth factor-related receptor levels | 26 | rs10191831 | T | C | -0.0591 | 0.4361 | 0.0125 | 2.27E-06 | 0.001799062 | 22.35037939 |
|  |  | rs10892466 | C | G | 0.0808 | 0.1236 | 0.0175 | 3.89E-06 | 0.001444964 | 21.31511405 |
|  |  | rs1133851 | A | G | -0.0664 | 0.2533 | 0.0142 | 2.92E-06 | 0.001528216 | 21.86244177 |
|  |  | rs116540722 | A | G | -0.1726 | 0.0313 | 0.0381 | 5.89E-06 | 0.00139084 | 20.51977905 |
|  |  | rs116794986 | A | G | 0.2476 | 0.9884 | 0.0554 | 7.85E-06 | 0.001353765 | 19.97205714 |
|  |  | rs116927267 | A | G | -0.1742 | 0.0257 | 0.0389 | 7.53E-06 | 0.001359207 | 20.05109725 |
|  |  | rs11725578 | C | G | -0.0584 | 0.697 | 0.0128 | 5.05E-06 | 0.001454897 | 20.81349222 |
|  |  | rs117343254 | C | G | -0.1281 | 0.942 | 0.0274 | 2.94E-06 | 0.001527539 | 21.85427149 |
|  |  | rs13107325 | T | C | 0.1066 | 0.0697 | 0.0233 | 4.76E-06 | 0.00141881 | 20.92876512 |
|  |  | rs194746 | T | C | 0.0528 | 0.4849 | 0.0112 | 2.43E-06 | 0.001507233 | 22.22147078 |
|  |  | rs207671 | A | G | 0.1016 | 0.5359 | 0.0117 | 3.83E-18 | 0.005250352 | 75.39714348 |
|  |  | rs2837988 | A | C | 0.0576 | 0.3578 | 0.0118 | 1.05E-06 | 0.001614686 | 23.82440114 |
|  |  | rs28689968 | T | C | 0.0918 | 0.1074 | 0.0184 | 6.07E-07 | 0.001686652 | 24.88804349 |
|  |  | rs35032874 | T | G | 0.132 | 0.7027 | 0.0132 | 1.52E-23 | 0.006950719 | 99.98600126 |
|  |  | rs62193248 | A | T | 0.213 | 0.6507 | 0.0123 | 3.50E-67 | 0.020558269 | 299.8390436 |
|  |  | rs62253189 | A | G | -0.108 | 0.9404 | 0.0244 | 9.59E-06 | 0.001329356 | 19.58884541 |
|  |  | rs6517655 | C | G | 0.0757 | 0.289 | 0.0129 | 4.41E-09 | 0.002404505 | 34.43115079 |
|  |  | rs7123587 | A | C | -0.0552 | 0.6266 | 0.0124 | 8.52E-06 | 0.001496536 | 19.81385988 |
|  |  | rs74890918 | A | G | 0.2266 | 0.0157 | 0.0502 | 6.36E-06 | 0.001424139 | 20.37284011 |
|  |  | rs75685422 | T | C | 0.0879 | 0.1139 | 0.0186 | 2.29E-06 | 0.001560747 | 22.33012024 |
|  |  | rs77209370 | A | C | 0.1346 | 0.9589 | 0.0298 | 6.28E-06 | 0.001431529 | 20.39842107 |
|  |  | rs773112 | T | C | 0.0566 | 0.3128 | 0.0125 | 5.95E-06 | 0.001433009 | 20.49991387 |
|  |  | rs7809355 | A | C | -0.0734 | 0.1553 | 0.0164 | 7.62E-06 | 0.001400087 | 20.02827859 |
|  |  | rs9289590 | T | C | -0.0652 | 0.7033 | 0.0142 | 4.40E-06 | 0.001696755 | 21.07892566 |
|  |  | rs9597690 | A | G | -0.0531 | 0.646 | 0.0118 | 6.80E-06 | 0.001373789 | 20.24724864 |
|  |  | rs9967959 | T | G | 0.0718 | 0.1718 | 0.0152 | 2.32E-06 | 0.001512419 | 22.3101631 |
| STAM binding protein levels | 18 | rs1034059 | A | G | 0.0695 | 0.698 | 0.0145 | 1.64E-06 | 0.00160533 | 22.97062484 |
|  |  | rs111581172 | A | G | -0.1722 | 0.0323 | 0.0352 | 9.98E-07 | 0.001621424 | 23.92886061 |
|  |  | rs116132562 | A | G | -0.1689 | 0.0333 | 0.0372 | 5.62E-06 | 0.001444848 | 20.61163526 |
|  |  | rs116198125 | T | C | -0.1484 | 0.9619 | 0.0325 | 4.97E-06 | 0.001413749 | 20.84692945 |
|  |  | rs12152184 | A | G | 0.0961 | 0.8851 | 0.0208 | 3.83E-06 | 0.001718231 | 21.34273458 |
|  |  | rs12531843 | C | G | -0.1251 | 0.9177 | 0.0243 | 2.63E-07 | 0.002132128 | 26.49915599 |
|  |  | rs138897138 | A | G | 0.1085 | 0.9138 | 0.0218 | 6.46E-07 | 0.001730704 | 24.76770084 |
|  |  | rs16847403 | T | C | 0.0584 | 0.7326 | 0.0131 | 8.27E-06 | 0.001348949 | 19.87119858 |
|  |  | rs2280441 | A | G | -0.1571 | 0.0404 | 0.0309 | 3.69E-07 | 0.001751153 | 25.84499544 |
|  |  | rs2584528 | T | G | 0.056 | 0.6302 | 0.0123 | 5.29E-06 | 0.001448654 | 20.72550089 |
|  |  | rs547553 | T | C | 0.2004 | 0.9798 | 0.0452 | 9.27E-06 | 0.001332894 | 19.65439072 |
|  |  | rs61962729 | T | G | -0.0978 | 0.9054 | 0.0217 | 6.58E-06 | 0.001419613 | 20.3094165 |
|  |  | rs6492915 | T | G | -0.1086 | 0.9115 | 0.024 | 6.04E-06 | 0.001734418 | 20.47215014 |
|  |  | rs73327656 | A | G | 0.235 | 0.0269 | 0.0512 | 4.44E-06 | 0.001524133 | 21.06361273 |
|  |  | rs73788059 | T | C | -0.092 | 0.0933 | 0.0204 | 6.49E-06 | 0.001380148 | 20.3355673 |
|  |  | rs7418913 | A | G | -0.0628 | 0.2712 | 0.0139 | 6.24E-06 | 0.001426587 | 20.40933674 |
|  |  | rs75610895 | T | C | -0.1908 | 0.0247 | 0.0394 | 1.28E-06 | 0.001589537 | 23.4479741 |
|  |  | rs851168 | A | G | 0.0616 | 0.3247 | 0.0136 | 5.91E-06 | 0.001601213 | 20.51236337 |
| **The causal effect of meniere's disease onset on inflammatory cytokines** | | | | | | | | | | |
| **Outcomes** | **nSNP** | **SNP** | effect_allele.outcome | other_allele.outcome | beta.outcome | eaf.outcome | se.outcome | pval.outcome | **R2** | **F** |
| Interleukin-10 levels | 13 | rs11140164 | T | C | -0.0014 | 0.2006 | 0.0154 | 0.928 | 6.33E-05 | 22.89258923 |
|  |  | rs114670060 | A | G | -0.0327 | 0.0116 | 0.0711 | 0.646 | 5.98E-05 | 21.64253376 |
|  |  | rs12952267 | A | G | 0.0396 | 0.2372 | 0.0167 | 0.0177 | 6.04E-05 | 21.8604982 |
|  |  | rs235887 | C | T | -0.0212 | 0.8221 | 0.0157 | 0.177 | 6.59E-05 | 23.83628861 |
|  |  | rs34925767 | G | A | -0.0464 | 0.0869 | 0.0214 | 0.0301 | 8.42E-05 | 30.47625816 |
|  |  | rs3777781 | A | T | 0.0145 | 0.2752 | 0.0131 | 0.268 | 8.51E-05 | 30.77939995 |
|  |  | rs62255912 | C | T | -0.0465 | 0.0366 | 0.0348 | 0.181 | 6.53E-05 | 23.6325262 |
|  |  | rs663494 | T | A | 0.0019 | 0.6282 | 0.0134 | 0.887 | 5.82E-05 | 21.05088801 |
|  |  | rs675645 | G | A | 0.0189 | 0.4661 | 0.012 | 0.115 | 5.89E-05 | 21.31257884 |
|  |  | rs72633289 | A | G | 0.0291 | 0.1084 | 0.0222 | 0.19 | 6.19E-05 | 22.41142517 |
|  |  | rs72762919 | T | C | 0.0038 | 0.1078 | 0.0197 | 0.847 | 5.90E-05 | 21.35065354 |
|  |  | rs72847949 | A | G | -0.0271 | 0.1409 | 0.0177 | 0.126 | 6.87E-05 | 24.87747109 |
|  |  | rs76320709 | A | C | 0.0381 | 0.0195 | 0.0479 | 0.426 | 5.77E-05 | 20.89355953 |
| Neurotrophin-3 levels | 13 | rs11140164 | T | C | -0.0076 | 0.2004 | 0.0153 | 0.619 | 6.33E-05 | 22.89258923 |
|  |  | rs114670060 | A | G | -0.1304 | 0.0119 | 0.0682 | 0.0559 | 5.98E-05 | 21.64253376 |
|  |  | rs12952267 | A | G | 0.0099 | 0.2373 | 0.0167 | 0.553 | 6.04E-05 | 21.8604982 |
|  |  | rs235887 | C | T | -0.0227 | 0.8222 | 0.0157 | 0.148 | 6.59E-05 | 23.83628861 |
|  |  | rs34925767 | G | A | -0.0313 | 0.0868 | 0.0214 | 0.144 | 8.42E-05 | 30.47625816 |
|  |  | rs3777781 | A | T | 0.0127 | 0.2749 | 0.0129 | 0.325 | 8.51E-05 | 30.77939995 |
|  |  | rs62255912 | C | T | -0.0442 | 0.0362 | 0.0345 | 0.2 | 6.53E-05 | 23.6325262 |
|  |  | rs663494 | T | A | 0.0086 | 0.6286 | 0.0134 | 0.521 | 5.82E-05 | 21.05088801 |
|  |  | rs675645 | G | A | -0.0174 | 0.4661 | 0.012 | 0.147 | 5.89E-05 | 21.31257884 |
|  |  | rs72633289 | A | G | 0.0138 | 0.1083 | 0.0222 | 0.534 | 6.19E-05 | 22.41142517 |
|  |  | rs72762919 | T | C | -0.0063 | 0.1079 | 0.0196 | 0.748 | 5.90E-05 | 21.35065354 |
|  |  | rs72847949 | A | G | 0.0132 | 0.1404 | 0.0174 | 0.448 | 6.87E-05 | 24.87747109 |
|  |  | rs76320709 | A | C | -0.0531 | 0.0195 | 0.0479 | 0.268 | 5.77E-05 | 20.89355953 |

***Supplementary material-figures***

Fig S1. Forest plots of Mendelian randomization analyses for CD40L receptor levels, Delta and Notch-like epidermal growth factor-related receptor levels, and STAM binding protein levels in MD.


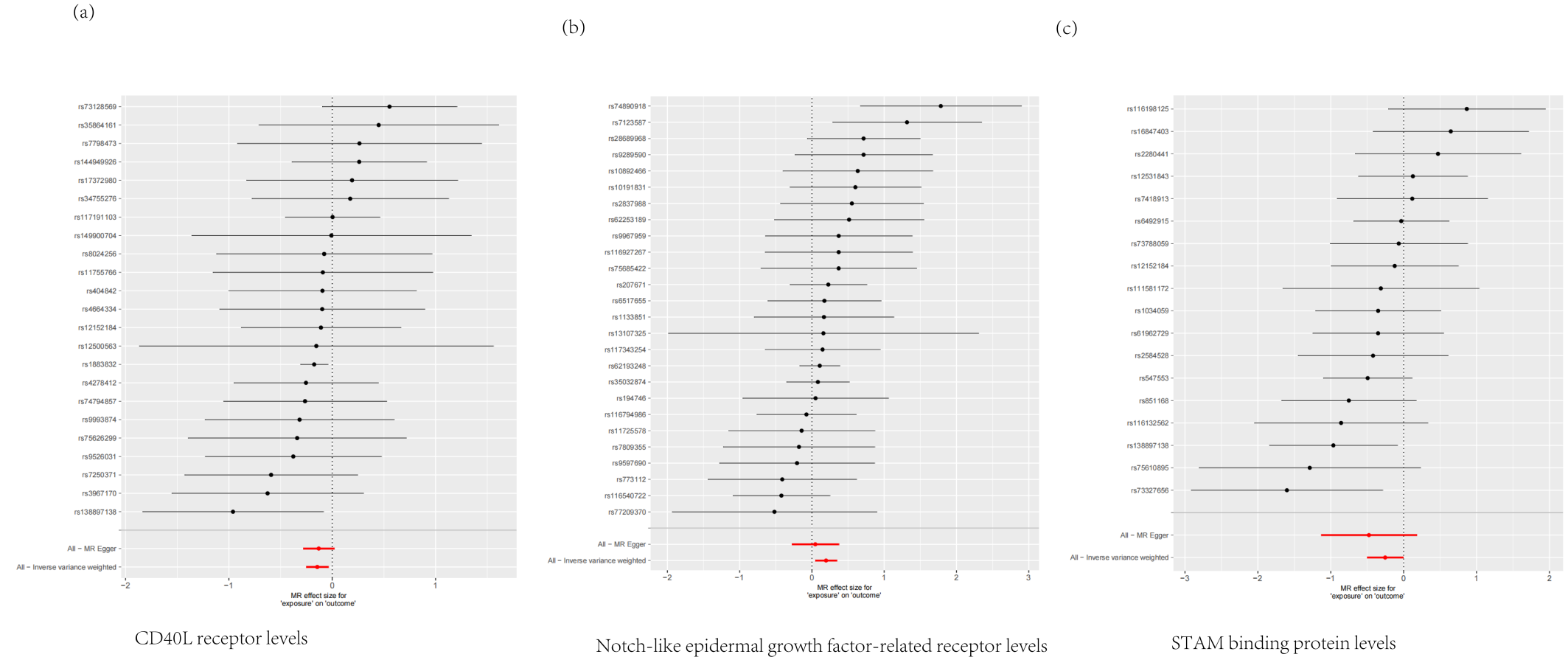


Fig S2. leave-one-out sensitivity analyses of Mendelian randomization analyses for CD40L receptor levels, Delta and Notch-like epidermal growth factor-related receptor levels, and STAM binding protein levels in MD.


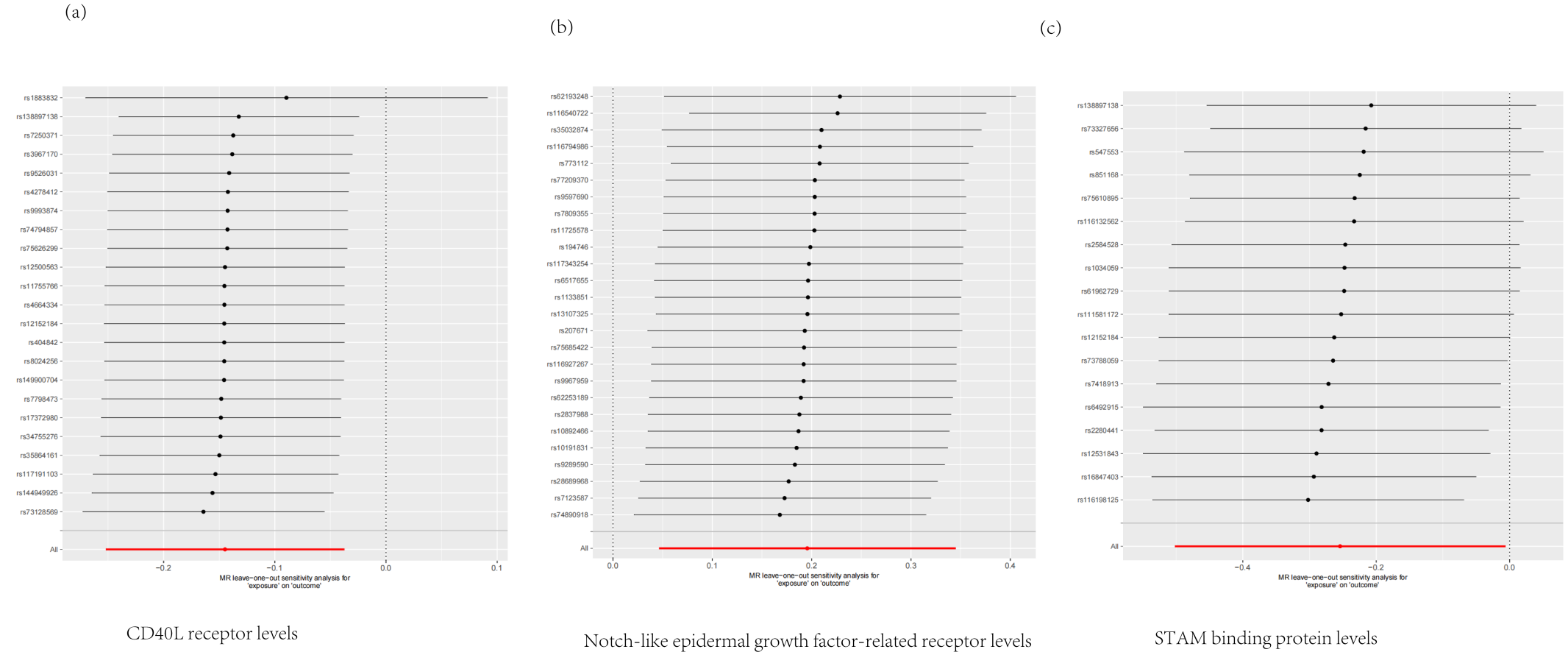


Fig S3. Forest plots of Mendelian randomization analyses between MD and inflammatory cytokines of Interleukin-10 levels and Neurotrophin-3 levels.


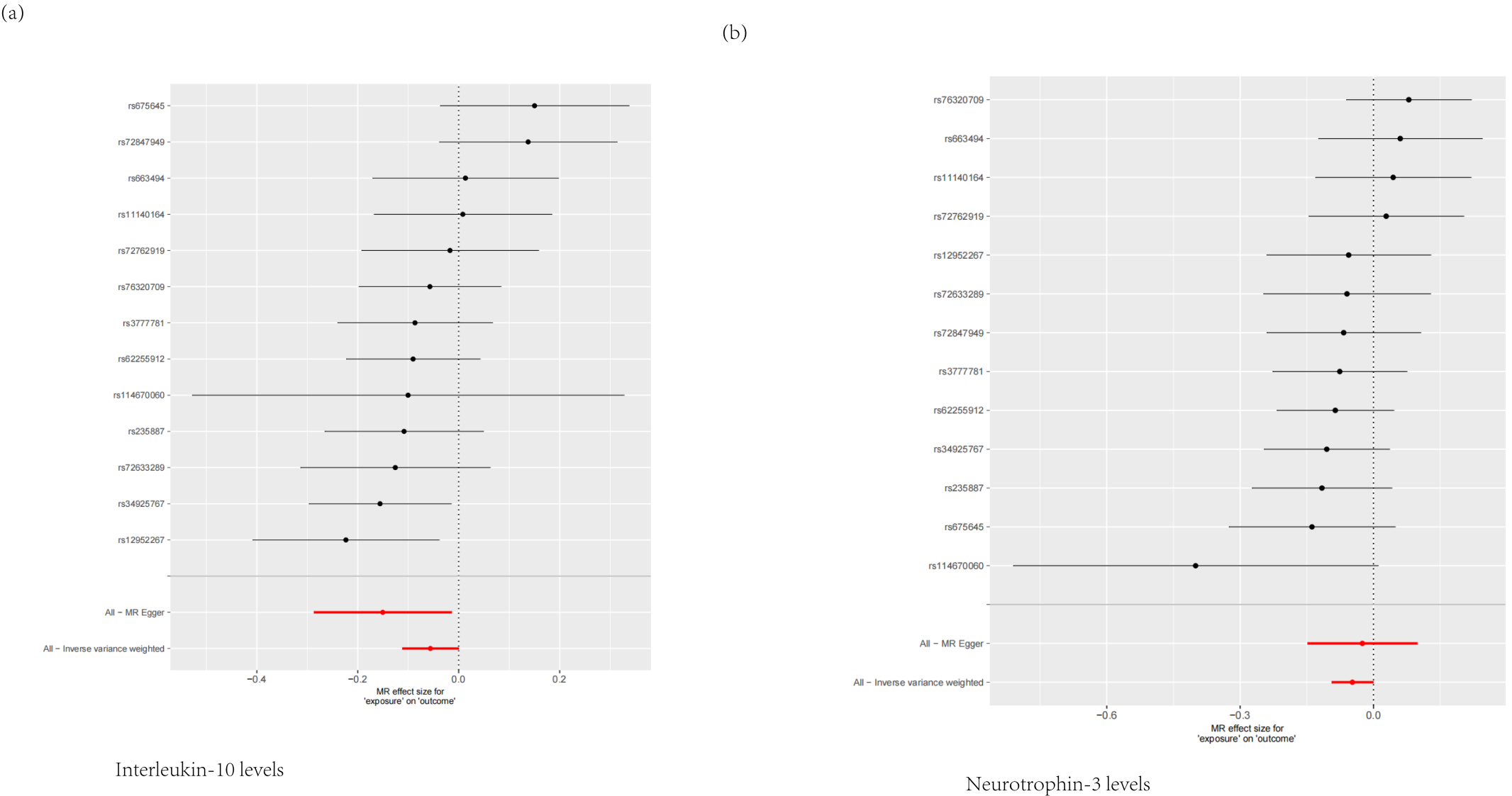


Fig S4. leave-one-out sensitivity analyses of Mendelian randomization analyses between MD and inflammatory cytokines of Interleukin-10 levels and Neurotrophin-3 levels.


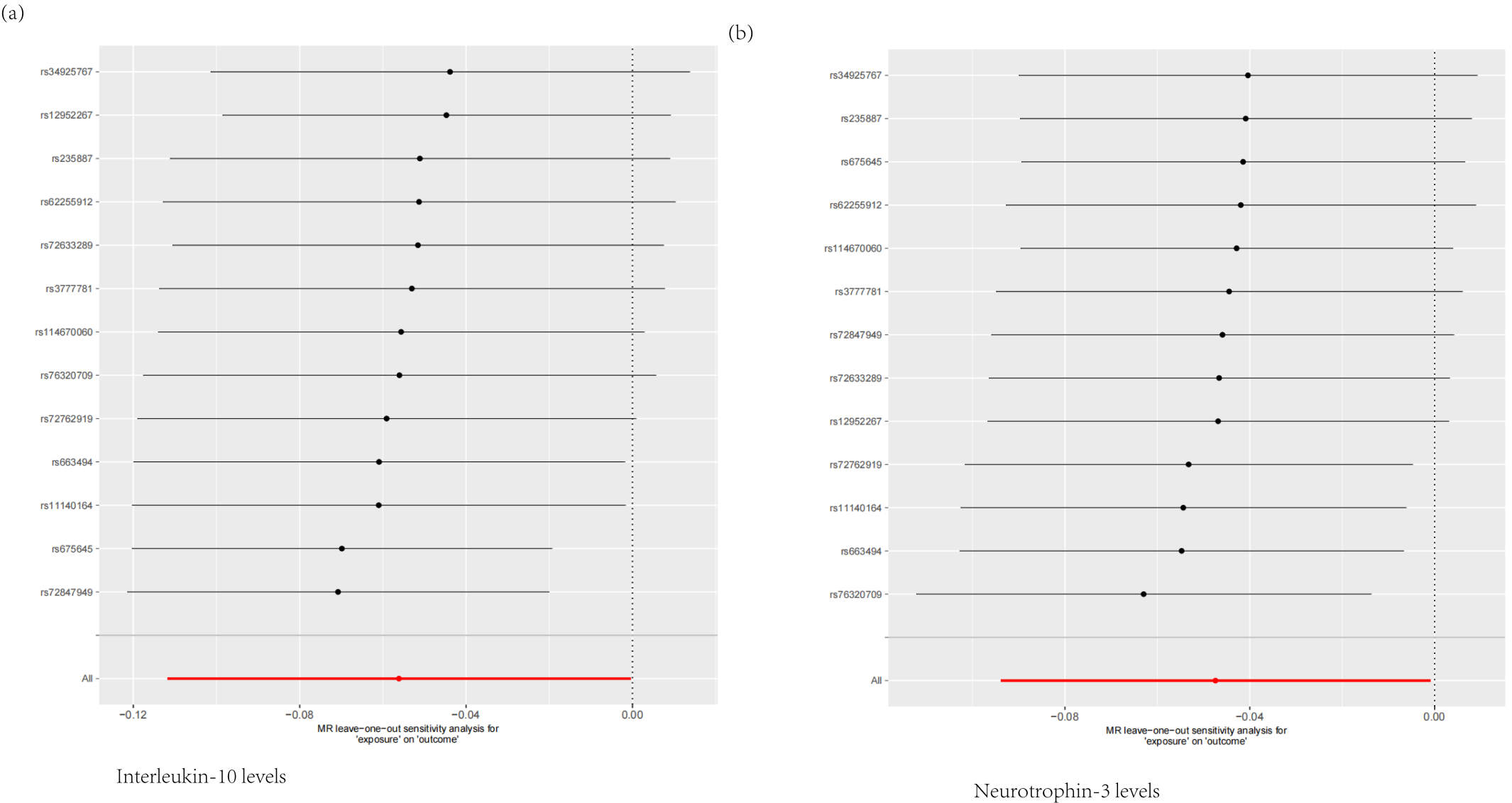

Supplement: Supplementary file 1 [file DataSheet_1.docx]
