# Supplementary material for: Exploring causal correlations between inflammatory cytokines and Ménière’s disease: a Mendelian randomization
Source: Front Immunol. 2024 Apr 29;15:1373723. doi: 10.3389/fimmu.2024.1373723 (PMC11089180; doi:10.3389/fimmu.2024.1373723)
Supplement: Supplementary file 2 [file Table_2.docx]

**STROBE-MR checklist of recommended items to address in reports of Mendelian randomization studies**^1^ ^2^

| **Item No.** | **Section** | **Checklist item** | **Page No.** | **Relevant text from manuscript** |
| --- | --- | --- | --- | --- |
| 1 | **TITLE and ABSTRACT** | Indicate Mendelian randomization (MR) as the study’s design in the title and/or the abstract if that is a main purpose of the study | 1 | A comprehensive two-sample Mendelian randomization (MR) analysis was conducted to determine the causal association between inflammatory cytokines and MD. |
|  | **INTRODUCTION** |  |  |  |
| 2 | **Background** | Explain the scientific background and rationale for the reported study. What is the exposure? Is a potential causal relationship between exposure and outcome plausible? Justify why MR is a helpful method to address the study question | 2 | Previous research has also closely linked cytokines such as IL-13 and IL-1β with MD. However, question of whether systemic inflammation is a cause or a consequence of MD remains subject to debate. This uncertainty arises from factors as disease progression, subsequent infection, medication usage after MD onset, and potential biases in observational studies due to unanticipated confounding variables or reverse causation. These complexities make it challenging to establish definitive causal relationships |
| 3 | **Objectives** | State specific objectives clearly, including pre-specified causal hypotheses (if any). State that MR is a method that, under specific assumptions, intends to estimate causal effects | 2 | In this study, we initially identified valid genetic variants from published genome-wide association study (GWAS) summary data of 91 inflammatory cytokines to investigate their correlations with MD. Subsequently, we examined the direction of causation by reversing the exposure and outcome. |
|  | **METHODS** |  |  |  |
| 4 | **Study design and data sources** | Present key elements of the study design early in the article. Consider including a table listing sources of data for all phases of the study. For each data source contributing to the analysis, describe the following: |  |  |
|  | a) | Setting: Describe the study design and the underlying population, if possible. Describe the setting, locations, and relevant dates, including periods of recruitment, exposure, follow-up, and data collection, when available. | 3 | The data for this MD analysis was sourced from FinnGen, a comprehensive public-private research project combining imputed genotype data from both newly collected and legacy samples from the Finnish biobank with digital health record data from the Finnish Health Registry. This integration aims to provide novel insights into disease genetics |
|  | b) | Participants: Give the eligibility criteria, and the sources and methods of selection of participants. Report the sample size, and whether any power or sample size calculations were carried out prior to the main analysis | 3 | MD Dataset comprised 2746 cases and 359,094 controls of European ancestry(<https://r9.risteys.finngen.fi/endpoints/H8_MENIERE>). The diagnostic criteria was ICD-10/H81.0. |
|  | c) | Describe measurement, quality control and selection of genetic variants | 3 | Selection of instrumental variables (IVs) section |
|  | d) | For each exposure, outcome, and other relevant variables, describe methods of assessment and diagnostic criteria for diseases | 3 | Immunity-wide GWAS data sources section |
|  | e) | Provide details of ethics committee approval and participant informed consent, if relevant | - |  |
| 5 | **Assumptions** | Explicitly state the three core IV assumptions for the main analysis (relevance, independence and exclusion restriction) as well assumptions for any additional or sensitivity analysis | 2 | MR analysis relies on three fundamental assumptions: exclusion restriction, independence, relevance (15). It is hypothesized that the identified genetic variants are linked to the risk factor (relevance) but not influenced by any confounding factors in the association between the risk factor and the outcome (independence). Furthermore, it is suggested that these variants solely affect the outcome through the specific risk factor of interest (exclusion restriction). In this two-way study, two GWASs were utilized to identify significant single nucleotide polymorphisms (SNPs) associated with 91 inflammatory cytokines and Ménière's disease. (Figure 1). |
| 6 | **Statistical methods: main analysis** | Describe statistical methods and statistics used |  |  |
|  | a) | Describe how quantitative variables were handled in the analyses (i.e., scale, units, model) | 4 | Initially, we established a genome-wide significant threshold (p <5 × 10-8) for selecting SNPs strongly related to MD and inflammatory cytokines. To address the limited available SNPs for cytokines as exposures, a more stringent cutoff (p <5 × 10-6) was initially employed. If an adequate number of SNPs were still not identified, the threshold was further adjusted. Ultimately, for our MR analysis, a threshold of p <1 × 10-5 was established.  The strength of the instruments was evaluated using the F-statistic to address potential biases arising from weak instruments |
|  | b) | Describe how genetic variants were handled in the analyses and, if applicable, how their weights were selected | 4 | Furthermore, to mitigate issues related to linkage disequilibrium, the identified SNPs were subjected to clumping (kb = 10,000, r2 = 0.001). |
|  | c) | Describe the MR estimator (e.g. two-stage least squares, Wald ratio) and related statistics. Detail the included covariates and, in case of two-sample MR, whether the same covariate set was used for adjustment in the two samples | 3,4 | MR Analysis section |
|  | d) | Explain how missing data were addressed | 4 | SNPs with an F-value below 10 were deemed weak instruments and were therefore excluded from the analysis. Finally, in instances where SNPs were unavailable in the outcome summary, they were substituted with proxy SNPs (R2 > 0.9) sourced from LDlink |
|  | e) | If applicable, indicate how multiple testing was addressed | - | - |
| 7 | **Assessment of assumptions** | Describe any methods or prior knowledge used to assess the assumptions or justify their validity | 3 | Furthermore, it is suggested that these variants solely affect the outcome through the specific risk factor of interest (exclusion restriction). In this two-way study, two GWASs were utilized to identify significant single nucleotide polymorphisms (SNPs) associated with 91 inflammatory cytokines and Ménière's disease. (Figure 1). |
| 8 | **Sensitivity analyses and additional analyses** | Describe any sensitivity analyses or additional analyses performed (e.g. comparison of effect estimates from different approaches, independent replication, bias analytic techniques, validation of instruments, simulations) | 4 | Pleiotropy and sensitivity analysis section and Reverse MR Analysis section |
| 9 | **Software and pre-registration** |  |  |  |
|  | a) | Name statistical software and package(s), including version and settings used | 4 | Statistical analyses were performed using specific R packages: TwoSampleMR (17) and MR-PRESSO. |
|  | b) | State whether the study protocol and details were pre-registered (as well as when and where) | - |  |
|  | **RESULTS** |  |  |  |
| 10 | **Descriptive data** |  |  |  |
|  | a) | Report the numbers of individuals at each stage of included studies and reasons for exclusion. Consider use of a flow diagram | 3 | Figure 2 |
|  | b) | Report summary statistics for phenotypic exposure(s), outcome(s), and other relevant variables (e.g. means, SDs, proportions) | 5 | (Supplementary Tables S1) |
|  | c) | If the data sources include meta-analyses of previous studies, provide the assessments of heterogeneity across these studies | - | - |
|  | d) | For two-sample MR:  i.  Provide justification of the similarity of the genetic variant-exposure associations between the exposure and outcome samples  ii.  Provide information on the number of individuals who overlap between the exposure and outcome studies | 5 | (Supplementary Tables S1) |
| 11 | **Main results** |  |  |  |
|  | a) | Report the associations between genetic variant and exposure, and between genetic variant and outcome, preferably on an interpretable scale | 5 | Supplementary Tables S1 |
|  | b) | Report MR estimates of the relationship between exposure and outcome, and the measures of uncertainty from the MR analysis, on an interpretable scale, such as odds ratio or relative risk per SD difference | 5,7 | **Figure 3,5** |
|  | c) | If relevant, consider translating estimates of relative risk into absolute risk for a meaningful time period | 5,7 | **Figure 3,5** |
|  | d) | Consider plots to visualize results (e.g. forest plot, scatter plot of associations between genetic variants and outcome versus between genetic variants and exposure) | 6,8 | **Figure4(a-f), figure6(a-d)** |
| 12 | **Assessment of assumptions** |  |  |  |
|  | a) | Report the assessment of the validity of the assumptions | 4 | No heterogeneity was detected in associations from three cytokine types, as evidenced by Cochrane's Q tests (both p-value of Q test from MR-Egger method and p-value of Q test from IVW method >0.05), while MR-PRESSO method identified no outlier SNPs. Additionally, the MR-Egger intercept disclosed no evidence of directional pleiotropy (p-value >0.05) |
|  | b) | Report any additional statistics (e.g., assessments of heterogeneity across genetic variants, such as *I^2^*, Q statistic or E-value) | 7 | Table 1 |
| 13 | **Sensitivity analyses and additional analyses** |  |  |  |
|  | a) | Report any sensitivity analyses to assess the robustness of the main results to violations of the assumptions | 7 | Table 1 |
|  | b) | Report results from other sensitivity analyses or additional analyses | 7 | MR-PRESSO Global Test p-value |
|  | c) | Report any assessment of direction of causal relationship (e.g., bidirectional MR) | 7 | Exploration of the causal effect of MD onset on inflammatory cytokines section |
|  | d) | When relevant, report and compare with estimates from non-MR analyses | - | - |
|  | e) | Consider additional plots to visualize results (e.g., leave-one-out analyses) | 7 | forest plots and leave-one-out sensitivity analyses of MR for the three cytokines in MD are in Supplementary Figure S1-S2. |
|  | **DISCUSSION** |  |  |  |
| 14 | **Key results** | Summarize key results with reference to study objectives | 5 | Our findings suggest that CD40L receptor, DNER, and STAM binding protein may serve as upstream contributors to MD. Conversely, when MD is considered the exposure in MR, it appears that it causally increase levels of IL-10 and Neurotrophin-3. We observed no reverse causality between any single biomarker and MD. It seems that several biomarkers might trigger MD onset, while others, such as certain inflammatory regulators, are likely implicated in disease progression. A comprehensive network model illustrating these relationships is presented in Figure 7. |
| 15 | **Limitations** | Discuss limitations of the study, taking into account the validity of the IV assumptions, other sources of potential bias, and imprecision. Discuss both direction and magnitude of any potential bias and any efforts to address them | 9 | several limitations should be acknowledged. Firstly, due to constraints in MR analysis, thorough examination of the second and third assumptions was not possible, potentially introducing biases. Secondly, the absence of individual data precluded further stratified population analyses. Thirdly, as the study relied on a European database, the findings may not be generalizable to other ethnic groups, thus limiting the applicability of our results. Finally, a looser threshold was used to evaluate results, which might have increased false positives but also allowed for a more comprehensive assessment of the strong associations between cytokines profiles and MD. Further research is warranted to validate our findings and to consider their application in clinical diagnostics and therapeutic strategies. |
| 16 | **Interpretation** |  |  |  |
|  | a) | Meaning: Give a cautious overall interpretation of results in the context of their limitations and in comparison with other studies | 5,7 | References 31-42 |
|  | b) | Mechanism: Discuss underlying biological mechanisms that could drive a potential causal relationship between the investigated exposure and the outcome, and whether the gene-environment equivalence assumption is reasonable. Use causal language carefully, clarifying that IV estimates may provide causal effects only under certain assumptions | 7 | Despite these discoveries, establishing causal connections between MD and key cytokines remain challenging due to limitations inherent in classical epidemiology. Classical epidemiological methods often enhance statistical power in identifying correlations but contribute minimally to theoretical understanding of causation (42). Interpreting a relationship between exposure and disease outcome in observational data as causal relies on assumptions that are often unattainable, such as the absence of unexpected confounding and reverse causation (43). Therefore, the observed increase in inflammatory cytokines among MD patients could signify various factors, including disease's cause, a treatment side effect, an underlying infection, or other concurrent pathological immune responses, distinctions not discernable in standard observational studies. Our cytokine MR study illuminates the immune system's close ties to EH in MD. |
|  | c) | Clinical relevance: Discuss whether the results have clinical or public policy relevance, and to what extent they inform effect sizes of possible interventions | 5 | The primary histopathological alteration observed in MD is EH...... |
| 17 | **Generalizability** | Discuss the generalizability of the study results (a) to other populations, (b) across other exposure periods/timings, and (c) across other levels of exposure | 9 | Thirdly, as the study relied on a European database, the findings may not be generalizable to other ethnic groups, thus limiting the applicability of our results. Finally, a looser threshold was used to evaluate results, which might have increased false positives but also allowed for a more comprehensive assessment of the strong associations between cytokines profiles and MD. |
|  | **OTHER INFORMATION** |  |  |  |
| 18 | **Funding** | Describe sources of funding and the role of funders in the present study and, if applicable, sources of funding for the databases and original study or studies on which the present study is based | 9 | This work was supported by Sichuan Health Care Commission project (Pro-ject No.Zh2020-102 of Sichuan Ganyan). |
| 19 | **Data and data sharing** | Provide the data used to perform all analyses or report where and how the data can be accessed, and reference these sources in the article. Provide the statistical code needed to reproduce the results in the article, or report whether the code is publicly accessible and if so, where | 9 | The datasets presented in this study can be found in online repositories. The names of the repository/repositories and accession number(s) can be found in the article/Supplementary Material. |
| 20 | **Conflicts of Interest** | All authors should declare all potential conflicts of interest | 9 | The authors declare that the research was conducted in the absence of any commercial or financial relationships that could be construed as a potential conflict of interest. |

This checklist is copyrighted by the Equator Network under the Creative Commons Attribution 3.0 Unported (CC BY 3.0) license.

1. Skrivankova VW, Richmond RC, Woolf BAR, Yarmolinsky J, Davies NM, Swanson SA, et al. Strengthening the Reporting of Observational Studies in Epidemiology using Mendelian Randomization (STROBE-MR) Statement. JAMA. 2021;under review.

2. Skrivankova VW, Richmond RC, Woolf BAR, Davies NM, Swanson SA, VanderWeele TJ, et al. Strengthening the Reporting of Observational Studies in Epidemiology using Mendelian Randomisation (STROBE-MR): Explanation and Elaboration. BMJ. 2021;375:n2233.
